# The subseafloor crustal biosphere: Ocean’s hidden biogeochemical reactor

**DOI:** 10.3389/fmicb.2024.1495895

**Published:** 2024-11-27

**Authors:** Alberto Robador

**Affiliations:** Department of Biological Sciences, University of Southern California, Los Angeles, CA, United States

**Keywords:** subseafloor biosphere, crustal microbiology, biogeochemical cycling, hydrothermal circulation, carbon productivity

## Abstract

Underlying the thick sediment layer in ocean basins, the flow of seawater through the cracked and porous upper igneous crust supports a previously hidden and largely unexplored active subsurface microbial biome. Subseafloor crustal systems offer an enlarged surface area for microbial habitats and prolonged cell residence times, promoting the evolution of novel microbial lineages in the presence of steep physical and thermochemical gradients. The substantial metabolic potential and dispersal capabilities of microbial communities within these systems underscore their crucial role in biogeochemical cycling. However, the intricate interplay between fluid chemistry, temperature variations, and microbial activity remains poorly understood. These complexities introduce significant challenges in unraveling the factors that regulate microbial distribution and function within these dynamic ecosystems. Using synthesized data from previous studies, this work describes how the ocean crustal biosphere functions as a continuous-flow biogechemical reactor. It simultaneously promotes the breakdown of surface-derived organic carbon and the creation of new, chemosynthetic material, thereby enhancing element recycling and ocean carbon productivity. Insights gained from the qualitative analysis of the extent of biogeochemical microbial activity and diversity across the temperature and chemical gradients that characterize these habitats, as reviewed herein, challenge traditional models of global ocean carbon productivity and provide the development of a new conceptual framework for understanding the quantitative metabolic potential and broad dispersal of the crustal microbial biome.

## Introduction

The biogeochemical role of microbes deeply buried beneath the seafloor is far more important than presumed possible 80 years ago (Zobell and Anderson, 1936; Zobell, 1938). Over the past decades, comprehensive studies of subseafloor sedimentary microbes have revealed not only cell abundances that match previous estimates in seawater and in surface sediments (Kallmeyer et al., 2012) but most importantly, have demonstrated the viability of these microbes (Morono et al., 2011; Trembath-Reichert et al., 2017; Imachi et al., 2019) and their essential role in operating and maintaining global biogeochemical cycles (Parkes et al., 2014). We now understand that beneath the sediment layer, fluids moving through the basaltic ocean crust hold a similar amount of organic carbon, stored within living prokaryotic biomass (~1.6 Gt C, Bar-On et al., 2018). The volume of the ocean crust biosphere represents nearly 2% of the volume of the oceans (Johnson and Pruis, 2003). Conditions along the active fluid flow paths that characterize this habitat indicate that the crustal biosphere is the most favorable of deep-subsurface habitats and is likely a very active site of element cycling (Johnson et al., 2006). Furthermore, this aquifer is hydrothermally active and interactions with the overlaying sediments and ocean seawater facilitate the free exchange of fluid, chemicals, biological material, and heat, which likely have a large impact on the variability of seawater chemical composition and global biogeochemical cycling (Edwards et al., 2011).

## The upper crustal reservoir as a sub-surface microbial biosphere

**Figure 1:**
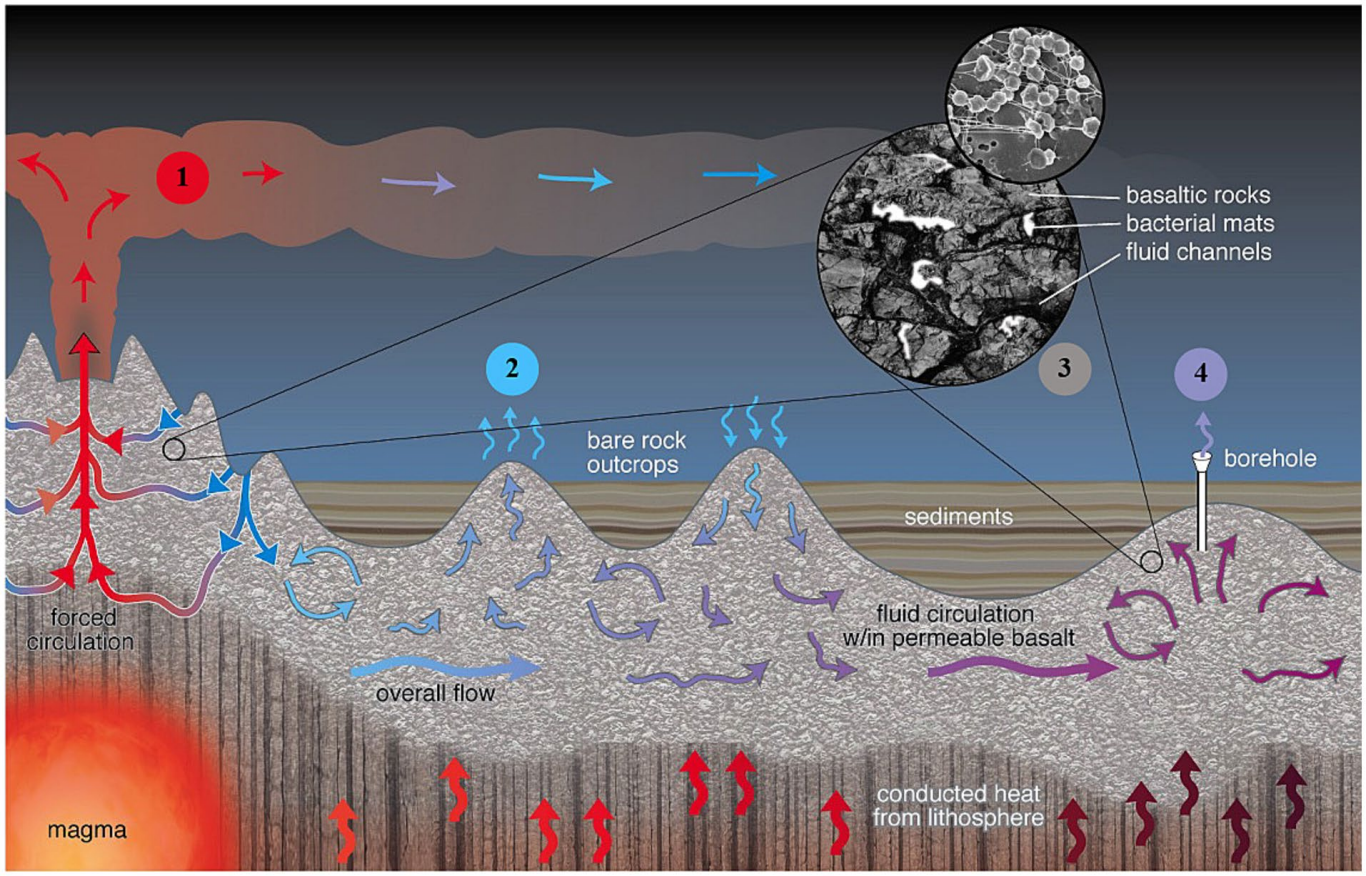
Several key access “windows” provide entry points to the crustal biosphere: ❶ Hydrothermal vents at mid-ocean ridges (MORs), featuring both diffuse and focused high-temperature flows (up to ~400°C) and event plumes associated with seafloor eruptions; ❷ Warm water seeps (<150°C) found at exposed rocky discharge zones on seamounts; ❸ Borehole cores extracted from sediment and basement rock layers; and ❹ Borehole CORK (Circulation Obviation Retrofit Kit) observatories, installed in boreholes, which offer the most precise control over sample placement, depth, and quality. These observatories provide unparalleled access to discrete depths in deep basement environments, enabling *in situ* sampling and experimentation with the highest degree of accuracy.

Basaltic ocean crust is formed at the axis of spreading mid-ocean ridges (MORs, Figure 1). As new ocean floor is formed and moves away from the spreading center, it is cooled by the interaction with seawater. Aging crustal porewaters remain generally isolated within buried upper oceanic basement, subjected to increasing temperatures as plates move away from spreading ridges. The accumulation of overlaying sediments on the MOR flanks and ocean basins prevents continued advective heat loss and results in strong hydrothermal gradients, which drive the rapid—on the order of m/day (Neira et al., 2016)—and largely lateral flow of low temperature (~5–65°C) fluids. Local circulation patterns are largely controlled by differences in pressure between cool (recharging) bottom seawater and warm (discharging) crustal fluids occurring at permeable igneous outcrops that penetrate the thick sediment cover (Winslow and Fisher, 2015; Winslow et al., 2016; Lauer et al., 2018). In contrast to the diffusion-dominated overlying sediments, the advective flow of hydrothermal fluids within the basaltic crust provides a pathway for the fast transport of solutes and particles including microbial cells, carbon and nutrients and generates small-scale variability in conditions supporting crustal biomes (Edwards et al., 2012a). The microbial biosphere, presumably located within the uppermost part of the igneous crust (Heberling et al., 2010), has likely been present since microbes first inhabited the oceanic crust around 3.5 billion years ago (Furnes et al., 2004). Quantitative knowledge of the extent of its metabolic potential and contribution to active global biogeochemical cycling, however, remains largely speculative (Orcutt et al., 2011b), as direct access to uncontaminated fluids in old ocean crust remains a major challenge.

## Access “windows” to crustal biosphere

The active circulation of hydrothermal fluids between open surfaces of the reservoir and overlying seawater provides multiple access “windows” to the subseafloor biosphere and, therefore, the opportunity to obtain high integrity samples from this challenging environment (Figure 1, #1–4). Most studies of subseafloor crustal microbiology (Figure 1, #1) have focused on MOR spreading areas, i.e., hydrothermal vents (Thorseth et al., 2001; Edwards et al., 2003; Lysnes et al., 2004), event plumes associated with seafloor eruptions (Meyer et al., 2013), and other settings where volcanism occurs (Fisk et al., 2003; Santelli et al., 2008). Active subseafloor microbial communities in venting fluids from MOR spreading axes are indicative of their local geologic setting and resultant geochemistry (Trembath-Reichert et al., 2019). These communities, however, do not reflect conditions in other crustal environments. The high-temperature focused fluid flows that characterized these MOR field sites tap the deepest and hottest (400°C) sub-crustal zone and can be considered end members with respect to the range of conditions that determine the nature of subseafloor crustal biomes (Elderfield et al., 1999). Most of the upper oceanic crust remains relatively cool, with temperatures below 150°C (Mottl and Wheat, 1994). Additionally, MOR flanks contribute more substantially to the global output of the upper crustal reservoir compared to the hotter axial regions, which play a comparatively minor role (Mottl, 2003). Recent research supports this disparity, showing that while axial zones release high-temperature fluids from hydrothermal activity near magma chambers, the ridge flanks exhibit a significantly greater overall heat flux through widespread low-temperature hydrothermal circulation (Urabe et al., 2015).

Exposed rocky discharge seamounts along MOR flanks in the other hand, are characterized by warm (<150°C) diffuse water seeps (Wheat et al., 2017) and offer an alternative access to the near surface basement biosphere (Figure 1, #2). Important surveys in exiting fluids of both heat and chemical fluxes as well as microbial communities have shown a high diversity of microorganisms in a continuum of near seafloor to deep subsurface biosphere communities (Hara et al., 2005; Huber et al., 2006; Lee et al., 2015). However, due in part to their generally diffuse flow there is a high mixing rate with overlying sediments (Meyers et al., 2014; Zinke et al., 2018) and, consequently, these sites have been studied to relatively limited extent.

At present, drilling represents the only access into the upper crustal reservoir for older, heavily sedimented crust (Figure 1, #3). Reports on the phylogenetic distribution of microbial communities within drilled marine basalts have revealed a cosmopolitan community with many members but vastly different from that in crustal fluids (Mason et al., 2007, 2009, 2010; Lever et al., 2013; Goordial et al., 2021). The extraction of borehole rock cores has no control over the placement, depth and quality of the samples (Lever, 2013), which leaves open the possibility of seawater or sediment contamination.

The best opportunities for co-located and simultaneous microbiological in situ sampling and experimentation have been provided by Borehole Circulation Obviation Retrofit Kit (CORK) observatories installed at Ocean Drilling Program (ODP/IODP) boreholes (e.g. Edwards et al., 2014; Fisher et al., 2005; Fisher et al., 2011) (Figure 1, #4). CORK’s critical attributes include the ability to penetrate through sediments into basement and isolate different horizons within the borehole, e.g., depth, degree of fracturing and differential flow rates. CORK observatories allow permanent access to samples through individual CORK installations as well as arrays of CORKs. Furthermore, *in situ* sampling and experimentation is possible using both downhole and seafloor fluid sampling systems. In downhole applications, flow-through osmotic pumping systems (FLOCS) represent an important technological advancement, providing a reliable fluid sampling solution. FLOCS are powerful steadfast osmotic pumps operating without any moving parts or electronics by bringing a reservoir of super-saturated salt solution to equilibrium (Orcutt et al., 2010; Edwards et al., 2012b). They are highly versatile and provide the pumping power to collect samples in manipulative experiments, such as colonization chambers that integrate various relevant mineral and control surfaces into the in-line flow path of the osmo-samplers (Orcutt et al., 2011a). On the other hand, technological advancements on seafloor applications, such as the GeoMICROBE sled (Cowen et al., 2012), represent significant progress in the ability to conduct long-term monitoring and in-depth analysis of microbial and chemical processes deep beneath the ocean floor. This innovative autonomous sensor and accompanying fluid sampling systems were designed to extract large volumes of fluid from crustal aquifers for surface analysis, highlighting a leap forward in crustal research capabilities (Lin et al., 2020). This article emphasizes investigations into the microbial community in the crustal subseafloor using borehole CORK observatories because they have yielded the most extensive knowledge on the crustal microbial biosphere (Orcutt et al., 2020) and stablished the foundation for present studies on the microbial community’s metabolic potential and role in global biogeochemical cycles.

## Emerging crustal microbiology

Microbial communities in crustal fluids exhibit spatial heterogeneity (Jungbluth et al., 2013) and inter-annual variability (Jungbluth et al., 2014) reflecting the dynamic and diverse environments that characterize the hydrogeological active upper igneous crust. Shared distinct microbial lineages, however, are consistent with the inferred hydrogeologic connectivity of these systems and the idea of permanent residency in the crustal subseafloor (Jungbluth et al., 2016). When analyzing datasets from different crustal habitats, the nature of the crustal environment (distinguished by either planktonic communities or mineral-attached communities), the prevailing redox conditions, and the geochemical evolution of these habitats collectively reveal patterns of global biogeographic distribution (Smith et al., 2016; Zhang et al., 2016b; Ramírez et al., 2019; Orcutt et al., 2021). Time-course studies of the genetic makeup and evolutionary trajectories of recovered metagenome-assembled genomes (MAGs) in subsurface crustal fluids (Anderson et al., 2022) have shown rapid allele frequency shifts linked to gene flow and recombination between microbial populations, which are mainly associated with stochastic events such as dispersal and mixing of populations throughout the aquifer. Despite this dynamism, however, temporal and spatial trends reconstructed from MAGs (Tully et al., 2018) have shown a significant amount of functional redundancy in the subseafloor microbial populations, which do not correspond to changes in the composition of the community over time. This implies global microbial functional stability in the ubiquitous crustal subseafloor biosphere.

Driven by geochemical redox gradients created by the continued interaction of seawater with the basaltic rock at different spatial and temporal scales, microbes in these crustal environments have developed physiological and metabolic strategies to exploit the specific conditions of their environment. In young, oxic, and low-temperature crustal environments –key to most global hydrothermal fluid circulation in the ocean—the metabolic reconstruction of recovered MAGs (Tully et al., 2018) has revealed microorganisms representative of heterotrophic and autotrophic lifestyles. These versatile microbes, however, are also poised to exploit hypoxic and anoxic conditions with alternative electron acceptors, i.e., nitrate and sulfate. Furthermore, complementary time-series metatranscriptomic data have confirmed that microbial communities in this environment are populated by motile mixotrophic and organotrophic bacteria active under both oxic and anoxic conditions (Seyler et al., 2021). In old, anoxic, and warm crustal environments –representative of situations that are common in all ocean basins where hydrothermal circulation is isolated from the deep ocean– the MAG of a versatile heterotrophic microbial population has been recovered capable of oxidizing reduced carbon species with nitrate, iron, and sulfur compounds as potential electron acceptors (Boyd et al., 2019). Comparative analysis of single-amplified genomes (SAGs) representative of other persistent lineages also suggests the heterotrophic potential of anaerobic crustal communities to respire organic carbon with sulfate and nitrate (Carr et al., 2019) and other external terminal electron acceptors such as iron and sulfur oxides consistent with the mineralogy of these environments (Booker et al., 2023). Further genomic evidence has also identified MAGs representative of chemosynthetic microbial communities residing in mineral biofilms (Smith et al., 2019). These communities are sustained by water-rock reactions in a process involving the use molecular hydrogen to convert inorganic carbon from seawater into organic matter, with additional energy supplementation derived from amino acids or peptides present in the mineral-attached biofilms (Smith et al., 2021). Altogether, this suggests the presence of highly dynamic microbial communities capable of rapid adaptation to varying redox conditions within the basaltic crustal fluids. Moreover, the transcription of genes related to biofilm formation and motility suggests an adaptation for spatial relocation in response to environmental shifts, crucial for accessing diverse nutrient and energy sources.

## The subseafloor crustal system as a continuous-flow biogeochemical reactor

The biogeochemical state of the subseafloor crustal reservoir is influenced by several factors. The age of the crust affects the geochemical extent of water-rock interaction, while the thermal state of the aquifer determines the nature and scale of metabolic reactions that can occur. Fluids along the flow paths of the hydrogeologically active upper oceanic crust exhibit a geochemical reaction consistent with a decline in the redox potential of oxidants and concentrations of organic material (Lin et al., 2012). By leveraging compositional geochemical data of basement fluids relative to bottom seawater, comprehensive thermodynamic modeling of diverse redox reactions has shown a geochemical shift toward lower free energy availability with increasing residence time and temperature (Robador et al., 2015). Despite this energetic constraint, direct heat measurements indicative of the change in enthalpy associated with total microbial activity have demonstrated the high metabolic potential of microbes to respire under oxic and anoxic conditions (Robador et al., 2016). This is observed in fluids characteristic of both young, oxygenated, and warm, highly reacted end-member areas of the upper oceanic crust, respectively. Furthermore, extensive studies have demonstrated the widespread potential of microbial activity in crustal fluids, with high rates of autotrophy and heterotrophy comparable to those found in surface environments (Meyer et al., 2016; Robador et al., 2016; Zhang et al., 2016a; Trembath-Reichert et al., 2021). These findings suggest that the upper crustal reservoir, functioning as a subsurface microbial biosphere, may be as metabolically active as the overlying ocean.

Additionally, the rate at which fluid flows through the crust influences the replenishment of reactants and the removal of byproducts, thus affecting the concentration of chemical species. During the geochemical evolution of fluids, redox species are removed, further shaping the chemical composition. This geochemical evolution of crustal fluids strongly suggests *in situ* microbial consumption within the basement (Orcutt et al., 2013; Robador et al., 2015). However, sediment pore water data from near sediment-basement interface (Elderfield et al., 1999) indicate that deep sediments must also serve as a sink for basement electron acceptors, which diffuse into the overlaying depleted sediments. This dynamic exchange supplies essential electron acceptors, such as oxygen and sulfate, to deeper sediment layers, which is crucial for sustaining microbial metabolisms within the sediment. Deep sediment porewater profiles demonstrate that oxygen diffuses upward from the underlying basalt and nitrate accumulates in the overlying sediments (Ziebis et al., 2012). This observation strongly implicates the process of nitrification as both an autotrophic sink of porewater oxygen and a source of nitrate, which, in turn, may support heterotrophy via denitrification where oxygen is absent and, potentially, autotrophic growth via methanogenesis. Furthermore, diffusion of sulfate from crustal fluids into overlying sediments, can form a transition zone where sulfate meets *in situ*-produced methane, thereby stimulating microbial heterotrophic respiration, i.e., anaerobic methane oxidation coupled to sulfate reduction (Engelen et al., 2008; Fichtel et al., 2012, 2015). These microbial processes, in turn, accelerate the breakdown of organic carbon deposits. Concurrently, the sediments supply electron donors, including dissolved organic carbon (DOC), to the basement ecosystem. Higher sediment pore water DOC likely diffuses into the basement, making it a sink for both seawater and sediment DOC. This reciprocal exchange results in the alteration of DOC compounds with respect to seawater (LaRowe et al., 2017) and underscores the complex interplay between geochemical processes and microbial life in deep subsurface crustal environments, driving both the degradation and the recycling of organic materials (Shah Walter et al., 2018; Lin et al., 2019). The ocean crustal biosphere, therefore, functions to simultaneously degrade surface-derived organic carbon and to export new, chemosynthetic material (McCarthy et al., 2011; Lin et al., 2015). As such, the ocean crustal biosphere acts as a biogeochemical reactor, facilitating the transformation and transport of elements through various states and locations. By breaking down organic carbon from surface sources and synthesizing new materials through chemosynthesis, it promotes element recycling and enhances ocean carbon productivity. Given that the entire volume of seawater is estimated to cycle through the ocean crust approximately every 100,000 years (Johnson and Pruis, 2003), this continuous-flow biogeochemical reactor plays a crucial role in driving the cycling of key elements, supporting diverse microbial communities, and sustaining essential ecological processes in the ocean. The complex interactions and metabolic activities within the ocean crustal biosphere underscore its critical role in maintaining the balance of both oceanic and global biogeochemical cycles.

## Conclusion

The subseafloor crustal system plays a crucial role in the biogeochemical cycling within mid-ocean ridge flanks by redistributing mass and energy between deep sediments and basement aquifers and back into the ocean. Although quantitative estimates of microbial contributions to global biogeochemical cycles are limited by difficult access and sparse sampling, the widespread metabolic potential of this biosphere suggests that incorporating the crustal system into current global biogeochemical models will be crucial for accurately representing the full ocean carbon cycle. The ocean crustal biosphere plays a dual role, capturing the intricate processes of carbon degradation and synthesis occurring beneath the ocean floor. Recognizing its impact on global biogeochemical cycles will allow for a more comprehensive understanding of carbon mineralization and recycling, enhancing the accuracy of predictions related to ocean productivity and ecological dynamics. Moreover, studying the ocean crustal biosphere has significant implications for astrobiology, as it serves as an analog for potential life on other planetary bodies (Jones et al., 2018). Understanding how microbial life thrives in these extreme conditions on Earth offers valuable insights into the potential for life in similar environments elsewhere in the solar system and beyond. This connection highlights the broader importance of studying these systems, not only to improve our biogeochemical models but also to advance our search for life beyond Earth.
